# Multidisciplinary Approaches and Molecular Diagnostics in New-Onset Refractory Status Epilepticus and Nontuberculous Mycobacterial CNS Infections in the ICU: A Case Report

**DOI:** 10.7759/cureus.76449

**Published:** 2024-12-26

**Authors:** Pedro Manuel Batarda Sena, Margarida Ferro, Joana Alves Cabrita, Carlos Pontinha, Ana Mestre, Mário Oliveira, Luis Bento

**Affiliations:** 1 Intensive Care Department, Hospital Central do Funchal, Funchal, PRT; 2 Neurology Department, Hospital de S. José, Unidade Local de Saúde de São José, Lisbon, PRT; 3 Intensive Care Unit, Unidade Local de Saúde de São José, Lisbon, PRT; 4 Pathology and Laboratory Medicine Department, Unidade Local de Saúde de São José, Lisbon, PRT; 5 Internal Medicine Department, Hospital Distrital de Santarém, Santarém, PRT; 6 Medical Emergency Unit, Unidade Local de Saúde de São José, Lisbon, PRT

**Keywords:** central nervous system, central nervous system infections, diagnostic tools, granulomatous meningitis, intensive care, intensive care unit, molecular diagnostics, molecular diagnostics multidisciplinary management granulomatous meningitis next-generation sequencing, new-onset refractory status epilepticus, nontuberculous mycobacterial infections

## Abstract

The diagnosis and management of complex neurological conditions such as New-Onset Refractory Status Epilepticus (NORSE) and central nervous system (CNS) infections caused by non-tuberculous mycobacteria (NTM) pose substantial difficulties in intensive care units (ICUs). This article combines a case report and a literature review that explores the diagnostic dilemmas and therapeutic strategies for these critical conditions.

We report the case of an 83-year-old female with chronic granulomatous meningitis secondary to NTM, presenting a challenging diagnostic and complex management complexity typical of such a rare disease through a period time of five years. Her case emphasized the importance of a multidisciplinary approach in such cases, as the interplay between neurology and intensive care was vital. The need for new molecular diagnostic technologies is shown to be of high significance in identifying the causative pathogens and improving patient outcomes in these rare but critical conditions.

## Introduction

The intensive care unit (ICU) is a critical environment where clinicians are often tasked with managing rare and severe conditions that defy straightforward diagnosis and treatment [1-3]. Among these, New-Onset Refractory Status Epilepticus (NORSE) and central nervous system (CNS) infections caused by non-tuberculous mycobacteria (NTM) represent two particularly challenging clinical entities. While uncommon, both are associated with high rates of morbidity and mortality, often exacerbated by delays in diagnosis and the limitations of traditional investigative methods. Their clinical presentations are diverse and can easily be mistaken for more prevalent neurological or infectious diseases, which further complicates timely recognition [1-3].

NORSE is a life-threatening neurological emergency characterized by status epilepticus that does not respond to standard therapies and arises without a clear precipitating cause [4,5]. Despite improvements in diagnostic capabilities, many cases remain cryptogenic, leaving clinicians without a clear path to targeted treatment and often resulting in unfavorable long-term outcomes [4,5]. On the other hand, NTM CNS infections, though rare, are increasingly identified as significant causes of chronic meningitis and brain abscesses [6,7]. These infections frequently mimic conditions such as tuberculosis or fungal diseases, leading to diagnostic uncertainty that delays appropriate interventions [1-3].

Despite their distinct pathophysiological underpinnings, these conditions share critical features that justify a combined discussion in this report. Both require advanced diagnostic tools and a multidisciplinary approach to optimize care [1,3,7]. Techniques such as next-generation sequencing (NGS) have transformed pathogen identification and autoimmune mechanism detection, offering hope for more precise and timely diagnoses [1,6]. Yet, their integration into routine practice remains limited by challenges, including cost, accessibility, and the need for specialized expertise [1,3].

This report seeks to shed light on the diagnostic and therapeutic complexities of NORSE and NTM CNS infections, illustrating their shared challenges through the case of a patient whose prolonged diagnostic journey underscores the necessity of a collaborative, innovative approach. By exploring these conditions side by side, we aim to highlight the intersections between critical care, advanced diagnostics, and multidisciplinary management, offering insights that could bridge existing gaps in knowledge and improve patient outcomes in these complex scenarios [1,2].

## Case presentation

**Figure 1:**
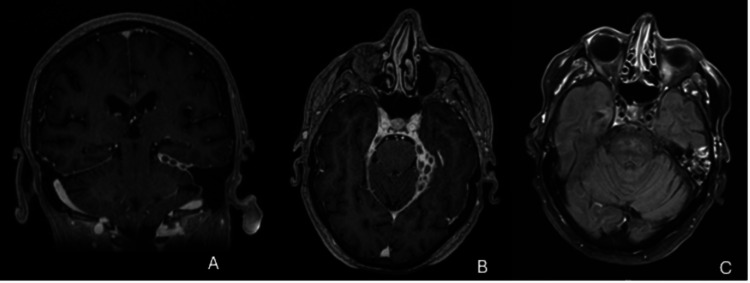
Coronal and axial navigation protocol cranioencephalic MRI (A/B) Coronal and axial navigation protocol cranioencephalic MRI showing a contrast-enhancing extra-axial lesion in the left cerebellar tentorium initially suspected as a multiloculated meningioma. (C) Axial T2-FLAIR cranioencephalic MRI highlights atrophic-gliotic changes in the left temporal region, interpreted as post-surgical sequelae. These findings were critical in ruling out alternative causes and identifying surgical targets.

In 2019, an 83-year-old woman presented with progressive diplopia due to right-sided right abducent nerve palsy. Imaging revealed a contrast-enhancing lesion in the cerebellar tentorium, initially suspected to be a meningioma (Figure 1), however. Surgical resection confirmed granulomatous inflammation. No definitive etiology was identified despite extensive testing, including polymerase chain reaction (PCR) for Mycobacterium tuberculosis and fungal pathogens. The case was classified as idiopathic granulomatous disease.

The patient remained clinically stable and asymptomatic for five years. However, in January 2024, she experienced recurrent motor focal seizures, aphasia, fluctuating levels of consciousness, and generalized neurological decline. These symptoms raised the suspicion of structural temporal epilepsy due to prior surgical sequelae. Despite initiating antiepileptic therapy antiseizure medication (ASM), the patient progressed to refractory non-convulsive status epilepticus (NCSE), requiring ICU admission.

**Figure 2:**
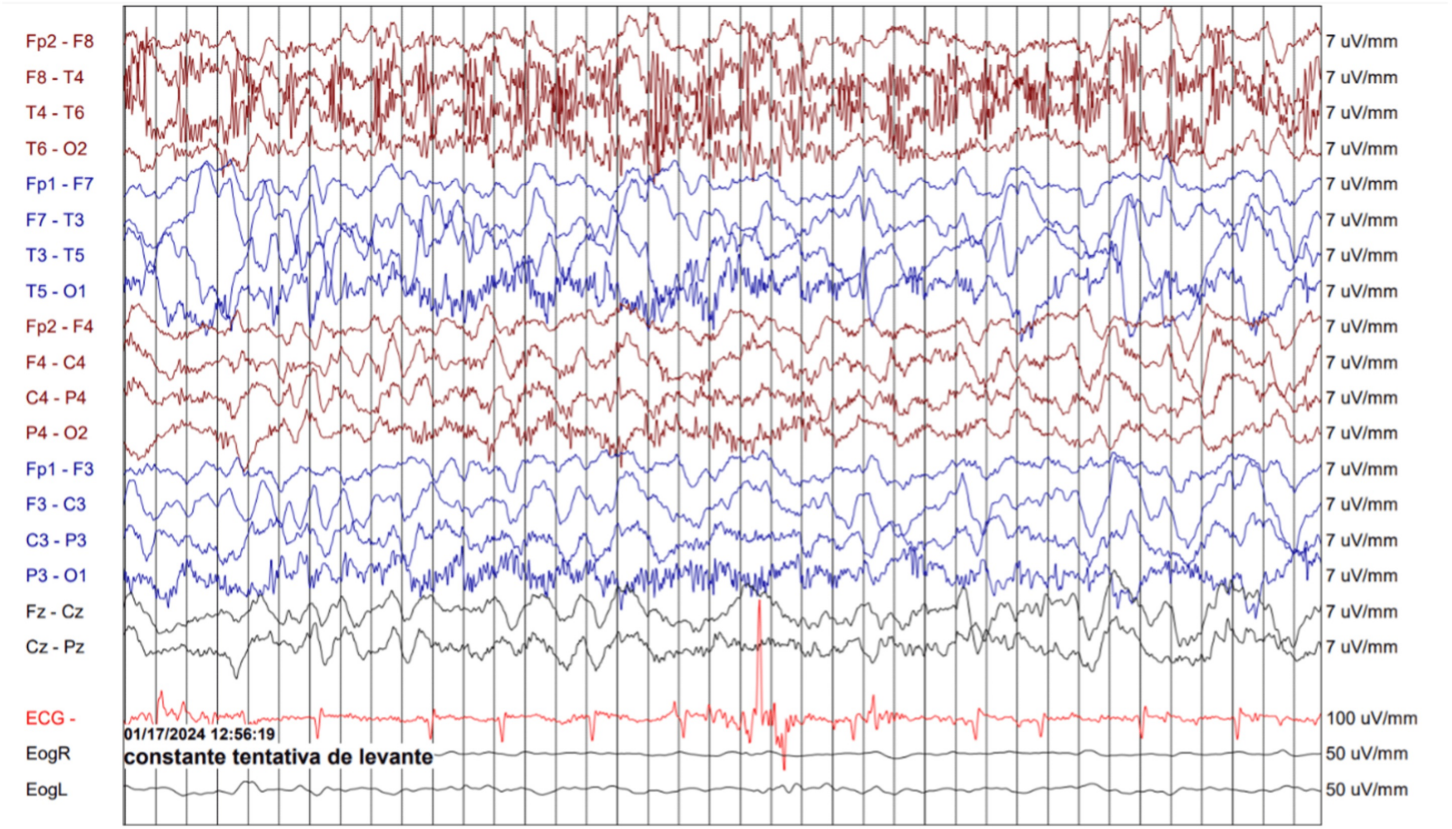
Electroencephalogram (EEG) using a 10-20 system bipolar montage showing slow background activity with left temporal Periodic Lateralized Epileptiform Discharges (PLEDs), supporting the diagnosis of non-convulsive status epilepticus in the appropriate clinical context

Electroencephalography (EEG) confirmed NCSE with left temporal periodic lateralized epileptiform discharges (PLEDs) (Figure 2).

In the ICU, the control of seizures required high-dose midazolam, propofol, and levetiracetam. Persistent NCSE required further adding lacosamide and valproate.

Magnetic resonance imaging (MRI) revealed post-surgical gliosis scarring without other abnormalities, and PCR and cultures for bacterial and viral pathogens were negative. Cerebrospinal fluid (CSF) analysis showed elevated protein levels (MD1) (107 mg/dl) with average glucose and no pleocytosis. PCR and cultures for bacterial and viral pathogens were negative.

Empirical broad-spectrum antibiotics and antivirals failed to alter the clinical course. Systemic complications happened, including a left femoral artery hematoma, requiring RBC transfusions, and adrenal insufficiency, managed with hydrocortisone replacement therapy. Those systemic complications highlighted the complexity of her ICU stay. Due to prolonged ventilatory support, tracheostomy placement was needed.

Due to a lack of clinical improvement and progressive neurological decline, archived specimens from the 2019 surgery were retrieved and subjected to next-generation sequencing (NGS). This analysis identified DNA from Mycobacterium, a rapidly growing non-tuberculous mycobacterium. The histopathological review further confirmed granulomatous inflammation with only two acid-fast bacilli staining. This finding marked a pivotal turning point in the diagnostic process, redefining the approach to her management.

A targeted antimycobacterial regimen consisting of rifampin, ethambutol, and clarithromycin was promptly initiated.

Subsequent weeks saw the patient demonstrate gradual neurological recovery. EEG monitoring confirmed NCSE resolution, and her consciousness level steadily improved. By the time of her transfer to the ward, she had demonstrated partial neurological recovery, including improved motor function, the ability to follow simple commands, and intermittent verbal interaction.

The patient’s diagnostic and therapeutic trajectory, spanning several years, is summarized in Table 1. This timeline underscores the diagnostic delays and evolving clinical challenges characteristic of NTM CNS infections, which often mimic other chronic CNS conditions [2,3,8]. It also highlights the critical role of specimen preservation and molecular diagnostics, such as NGS, in achieving a definitive diagnosis [1,3].

**Table 1:** Case timeline

Year/Date	Event	Outcome/Findings
2019	Initial presentation with diplopia due to abducent palsy	Lesion suspected as meningioma, resected
2019	Histopathology showed granulomatous inflammation	Diagnosis classified as idiopathic granulomatous disease
January 2024	New symptoms: seizures, aphasia, consciousness fluctuation	Intensive care unit admission for refractory non-convulsive status epilepticus, considered to be structural epilepsy secondary to surgical sequelae.
February 2024	Electroencephalogram confirmed non-convulsive status epilepticus; Blood laboratory analysis, cerebrospinal fluid analysis, and imaging studies were non-revealing.	Request for the archived specimen from 2019
March 2024	Archived specimen re-analyzed with next-generation sequencing	Nontuberculous mycobacteria deoxyribonucleic acid identified; treatment adjusted
April 2024	Intensive care unit management with targeted antimicrobials	Gradual neurological improvement

Key diagnostic challenges and turning points

Initial Diagnosis

The initial misclassification as idiopathic granulomatous disease, despite comprehensive PCR testing, delayed targeted treatment.

Progressive Symptoms in 2024

Recurrent seizures and NCSE presented diagnostic uncertainty, as conventional investigations, including imaging and CSF studies, failed to yield conclusive results.

NGS Breakthrough

The identification of Mycobacterium DNA via NGS underscored the necessity of advanced molecular diagnostics in managing elusive CNS infections.

Multidisciplinary Management

The interplay between neurologists, intensivists, pathologists, and infectious disease specialists facilitated the integration of diagnostic findings into a tailored therapeutic regimen.

## Discussion

This case underscores the intricate challenges in diagnosing and managing CNS infections caused by NTM, a rare but significant etiology in critically ill patients. Granulomatous meningitis due to NTM frequently mimics more common conditions, such as tuberculosis or fungal infections, often leading to misdiagnoses or delays in initiating targeted treatment [1,2]. This overlap is heightened by the protean manifestations of NTM infections, ranging from chronic meningitis to mass lesions, depending on pathogen virulence and host immune status [3].

ICU management posed additional challenges, as refractory NCSE required aggressive seizure control with sedatives, antiepileptic drugs, and prolonged EEG monitoring (NORSE). Systemic complications, including hemorrhagic shock and adrenal insufficiency, demanded a multidisciplinary approach to optimize outcomes [2,4]. Coordinated efforts among intensivists, neurologists, infectiologists, and pathologists were instrumental in achieving a definitive diagnosis and tailoring the therapeutic approach [1,3].

In Table 2, we summarize the clinical features, epidemiology, and management strategies of NORSE and NTM CNS infections to contextualize their diagnostic and therapeutic challenges. As recent literature highlights, these conditions demand tailored approaches due to their diverse presentations and overlapping manifestations with other diseases [1-9].

**Table 2:** Comparison of NORSE and NTM CNS infections NORSE: New-onset refractory status epilepticus; NTM: Nontuberculous mycobacteria

Feature	New-onset refractory status epilepticus (NORSE)	Nontuberculous mycobacteria central nervous system (NTM CNS) infections
Epidemiology	20% of all status epilepticus (SE) cases; cryptogenic in most cases	Rare, increasing in immunocompromised and immunocompetent patients
Clinical Presentation	Recurrent seizures progressing to refractory status epilepticus; periodic lateralized epileptiform discharges observed on electroencephalogram.	Chronic meningitis, abscesses, nonspecific neurological symptoms
Diagnostic Challenges	Cryptogenic etiology, extensive testing often inconclusive	Mimics tuberculosis or fungal infections, limited sensitivity of traditional methods
Key Imaging Findings	Electroencephalogram: Burst suppression pattern and periodic lateralized epileptiform discharges; Magnetic resonance imaging: Non-specific changes	Hydrocephalus, mass lesions, meningeal enhancement
Management Strategies	High-dose anesthetic agents, antiepileptic drugs, and immunomodulatory therapies	Antimycobacterial therapy, prolonged treatment (≥12 months)

Table 3 provides a comprehensive overview of the diagnostic tools used for NORSE and NTM CNS infections. NGS has significantly improved pathogen detection, particularly in cases where conventional microbiological tests fail [1,3,6]. However, logistical and economic barriers, including high costs, limited availability, and the need for specialized expertise, hinder their integration into clinical practice [3,8].

**Table 3:** Diagnostic methods and their utility

Test	Utility	Limitations
Electroencephalogram	Detects periodic lateralized epileptiform discharges and non-convulsive status epilepticus	Non-specific for underlying causes
Magnetic resonance imaging	Identifies structural abnormalities	Limited in determining specific etiology
Cerebrospinal fluid analysis	Highlights protein/glucose changes	Low sensitivity for nontuberculous mycobacteria
Next-generation sequencing	Precise pathogen identification	High cost, limited availability
Polymerase chain reaction	Pathogen-specific testing	False negatives for slow-growing organisms

**Figure 3:**
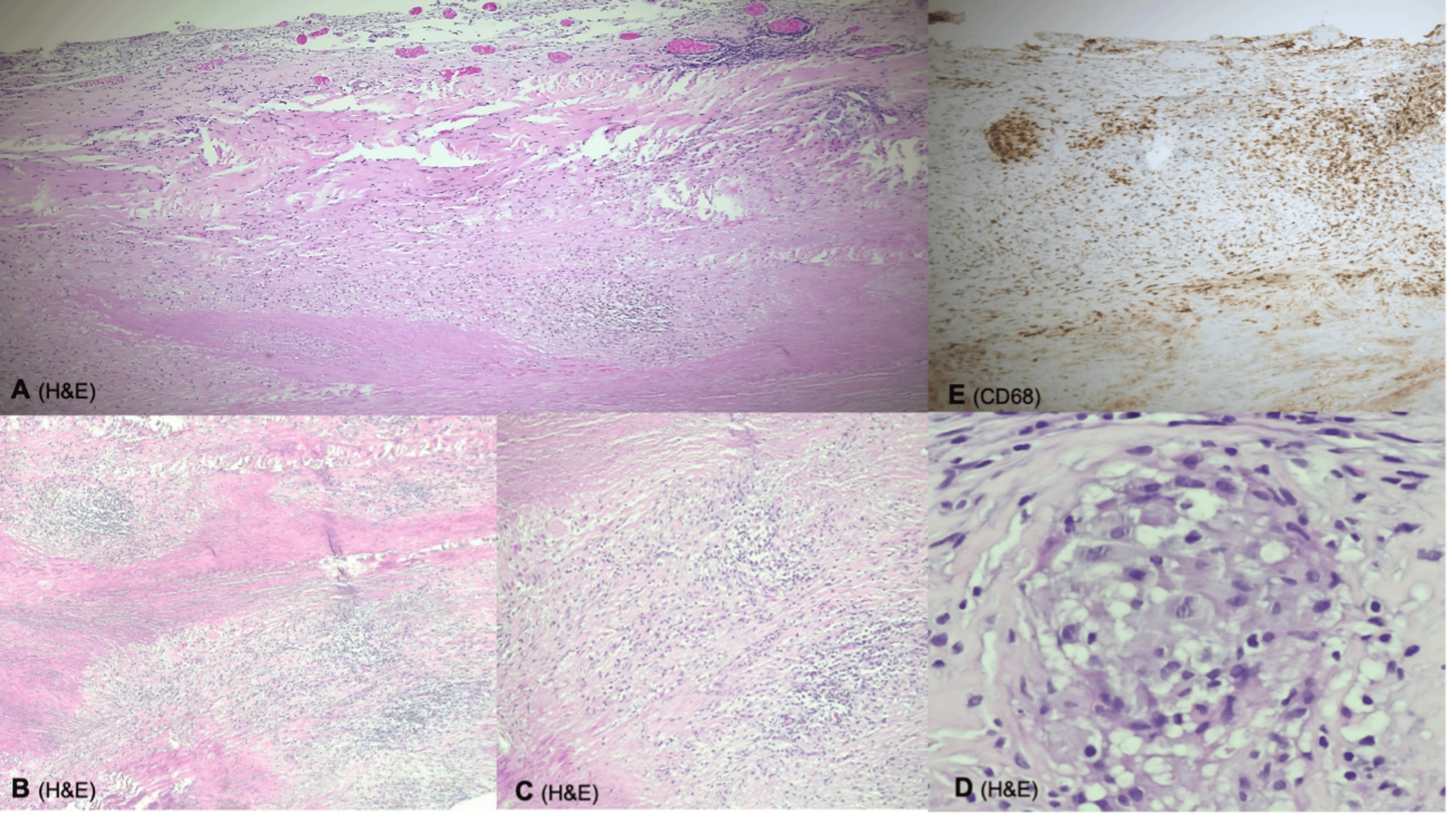
Histopathological examination of the dura mater showing granulomatous inflammation with necrotic foci. (A-D) Granulomas composed of histiocytoid cells (CD68+/CD1a-/Factor XIIIa-). (E) Inflammatory infiltrate predominantly consisting of CD3+ lymphocytes

In this specific case, the prolonged diagnostic delay, spanning over five years, proves the limitations of conventional diagnostic methods listed in Table 3, particularly in cases of slow-growing or atypical pathogens, and shows the importance of advanced microbiological and molecular testing to confirm the diagnosis [1,2]. The identification of NTM through NGS set a breaking point in the patient's clinical course. The medical team found the preservation and reanalysis of archived specimens to be pivotal, making clear the importance of robust specimen retention protocols and inter-hospital collaboration (Figure 3) [1,9].

The therapeutic approaches for refractory NCSE and NTM CNS infections in the ICU are detailed in Table 4. Managing these conditions requires a balance of prompt treatment and mitigating systemic complications, as described in guidelines for status epilepticus and chronic CNS infections [2,4,7,9]. Multidisciplinary collaboration is essential to optimize outcomes in such complex cases [5,6].

**Table 4:** ICU management of refractory non-convulsive status epilepticus  and nontuberculous mycobacteria central nervous system infections

Condition	Primary Therapies	Supportive Measures
Refractory non-convulsive status epilepticus	High-dose anesthetic agents and antiepileptic drugs	Electroencephalogram monitoring and management of complications.
Nontuberculous mycobacteria central nervous system infections	Rifampin, ethambutol, clarithromycin	Ventilatory support, corticosteroids

After proper diagnosis, the patient underwent a targeted antimycobacterial regimen comprising rifampin, ethambutol, and clarithromycin, and her medical status improved during the following weeks, proving once more the importance of molecular testing to properly improve the outcome of this disease.

## Conclusions

This case demonstrates the critical role of advanced molecular diagnostics, multidisciplinary collaboration, and integration of neurology and ICU expertise in managing rare CNS infections. Standardized protocols for specimen retention and expanded access to advanced molecular diagnostics, such as NGS, are pivotal for achieving better outcomes in such complex cases. Future initiatives should focus on enhancing accessibility to these technologies and developing robust evidence-based protocols to improve patient care.
